# Diagnosing lagophthalmos using artificial intelligence

**DOI:** 10.1038/s41598-023-49006-3

**Published:** 2023-12-08

**Authors:** Leonard Knoedler, Michael Alfertshofer, Siddharth Simon, Lukas Prantl, Andreas Kehrer, Cosima C. Hoch, Samuel Knoedler, Philipp Lamby

**Affiliations:** 1 Department of Plastic, Hand and Reconstructive Surgery, University Hospital Regensburg, Franz-Josef-Strauss-Allee 11, 93053 Regensburg, Germany; 2 Division of Hand, Plastic and Aesthetic Surgery, Ludwig-Maximilians-University Munich, Munich, Germany; 3 Northeastern University, Boston, MA USA; 4 Department of Otolaryngology, Head and Neck Surgery, School of Medicine, Technical University of Munich (TUM), 81675 Munich, Germany

**Keywords:** Medical research, Translational research

## Abstract

Lagophthalmos is the incomplete closure of the eyelids posing the risk of corneal ulceration and blindness. Lagophthalmos is a common symptom of various pathologies. We aimed to program a convolutional neural network to automatize lagophthalmos diagnosis. From June 2019 to May 2021, prospective data acquisition was performed on 30 patients seen at the Department of Plastic, Hand, and Reconstructive Surgery at the University Hospital Regensburg, Germany (IRB reference number: 20-2081-101). In addition, comparative data were gathered from 10 healthy patients as the control group. The training set comprised 826 images, while the validation and testing sets consisted of 91 patient images each. Validation accuracy was 97.8% over the span of 64 epochs. The model was trained for 17.3 min. For training and validation, an average loss of 0.304 and 0.358 and a final loss of 0.276 and 0.157 were noted. The testing accuracy was observed to be 93.41% with a loss of 0.221. This study proposes a novel application for rapid and reliable lagophthalmos diagnosis. Our CNN-based approach combines effective anti-overfitting strategies, short training times, and high accuracy levels. Ultimately, this tool carries high translational potential to facilitate the physician’s workflow and improve overall lagophthalmos patient care.

## Introduction

Lagophthalmos is broadly defined as the incomplete or abnormal closure of the eyelids with permanent widening of the palpebral fissure. Typically, three main types may underlie such inability to blink and/or close the eyes: (1) cicatricial (CL), (2) nocturnal (NL), and (3) paralytic lagophthalmos (PL)^1^.

Under healthy conditions, full eyelid closure is essential for maintaining a stable tear film and a moistened ocular surface. In patients suffering from CL, NL, and PL, the eye is not adequately wetted with tear fluid and, therefore, dries out. Such xerophthalmia and prolonged corneal exposure can trigger keratopathy and keratitis, ultimately progressing to corneal ulceration, reduction of visual acuity, or even blindness^2,3^. In order to preemptively avoid such sequelae, lagophthalmos must be diagnosed at an early stage and immediate targeted therapy should be initiated^4^.

The clinical-therapeutic armamentarium is broad and typically starts with conservative treatments in the form of lubricant drops/ointments, moisturize-retention chamber goggles, scleral contact lenses, or punctum plugs (which increasing the tear quantity by blocking the lacrimal drainage system)^5–8^. Off-label, despite the risk of paralyzing the superior rectus muscle and antagonizing the protective Bell’s phenomenon, Botolinumtoxin A may be injected off-label into the levator palpebrae superioris muscle and induces temporary ptosis lasting six to nine weeks^9,10^. In addition, central and lateral tarsorrhaphy represent surgical procedures aimed to fuse lid margins and narrow the horizontal and vertical palpebral aperture^11,12^. In elderly patients with refractory lagophthalmos, weight implants can be inserted into the upper eyelid (referred to as “lid loading”)^13^.

Across the variety of therapeutic options available and in light of impending complications of defective eye closure, timely and reliable detection of lagophthalmos remains imperative. In this context, algorithm-based tools with automated diagnostics would offer a wide variety of benefits. Specifically, (1) no expert knowledge is required, allowing the diagnosis to be established by non-medical staff and/or the patients themselves, (2) the time period from the onset of lagophthalmos to detection and treatment can be shortened, thereby preventing serious sequelae, and (3) in ambiguous patient cases, the suspected clinical diagnosis can be substantiated or refuted. Herein, we present an innovative method that leverages still-image processing to identify visual patterns via convolutional neural network technology and, ultimately, diagnose lagophthalmos.

## Results

### General training and testing accuracy

**Figure 1:**
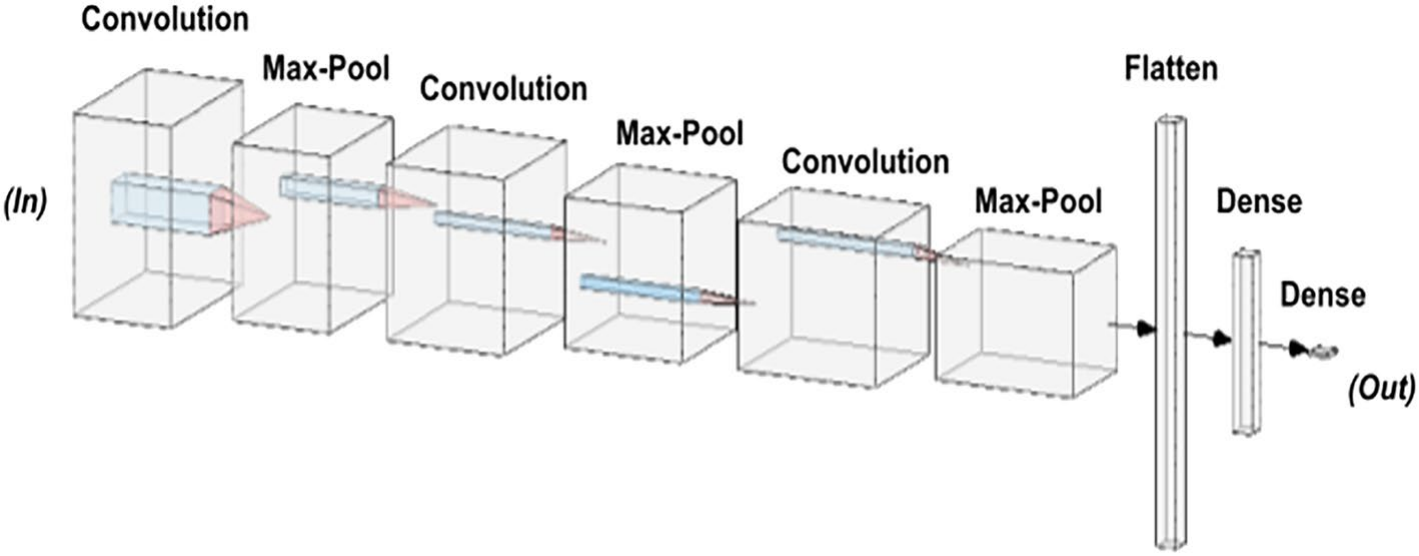
Layer visualization of the CNN. Initially, the data is filtered using multiple layers (i.e., “Convolution” and “Max-Pool”) followed by flattening strategies (i.e., combining multiple layers into one layer) and dense coding (i.e., condensing data information).

The presented model exhibited remarkable performance in terms of training, validation, and testing accuracy, achieving an average and final training accuracy of 85.8% and 91.2%, as well as an average and final validation accuracy of 88.2% and 97.8% over the span of 64 epochs. For training and validation, an average loss of 0.304 and 0.358 and a final loss of 0.276 and 0.157 were noted, respectively (Table 1). The model’s validation precision was noted to be 1.0000, and the recall was 0.9412, resulting in an F1 score of 0.9697. The model’s validation specificity was observed to be 1.0000, while its Area Under the Receiver Operating Characteristic Curve (AUROC) was noted to be 0.9977. The final model architecture achieved an accuracy of 93.41% and a loss of 0.2209 when classifying the testing set. The AUROC for the testing set was noted to be 0.9627, while the specificity was noted to be 0.9844; moreover, the model displayed a recall of 0.8148 and a precision of 0.9565. The model was trained for 17.3 min, during which the accuracy metrics demonstrated a consistent increase while the associated losses showed a steady decrease. These results indicate a progressive enhancement of the model's ability to correctly classify both training and testing data instances (Fig. 1).

**Table 1:** Model performance summary.

	Validation	Training	Testing
Final accuracy	97.8%	91.2%	93.4%
Final loss	0.157	0.276	0.221
Average accuracy	88.2%	85.8%	N/A
Average loss	0.304	0.358	N/A

### Influence of number of epochs on training and validation accuracy

**Figure 2:**
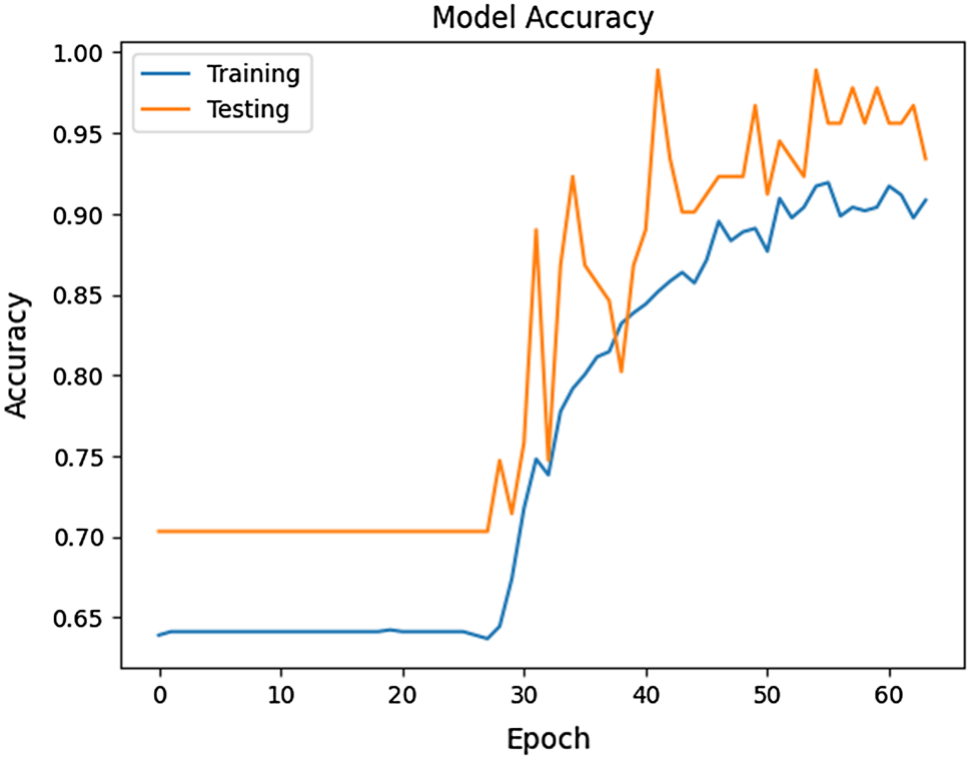
Model training and validation over 64 epochs.

The model's validation accuracy reached its peak at the 42nd epoch, while the training accuracy reached its peak at the 56th epoch, suggesting the model's continued learning and refinement with each epoch. Interestingly, the model showcased robust diagnostic capabilities even when presented with partially open eyelids, suggesting the model’s ability to effectively recognize and classify relevant features despite potential variations in the presentation of the input data (Fig. 2). Throughout most epochs, the training accuracy remained slightly lower than the validation accuracy, indicating that the model generalizes well to unseen data. However, an exception occurred during the 39th epoch, where the training accuracy achieved 83.2%, surpassing the training accuracy, which stood at 80.2% (Fig. 2).

## Discussion

The integration of software applications in patient medical care has witnessed significant growth in recent years, with various mobile health (mHealth) solutions being developed to support the diagnosis and management of different conditions^14–18^. Our study contributes to this evolving field by introducing a novel application for comprehensive lagophthalmos diagnosis, leveraging the power of convolutional neural networks (CNN) to analyze images of periocular regions.

One of the notable achievements of our study is the ability to avoid overfitting despite the relatively small training dataset of 917 images. Overfitting occurs when a model becomes too specialized in capturing patterns from the training data, leading to poor generalizability on unseen data. Our CNN demonstrated an impressive validation accuracy of 97.8%, surpassing the training accuracy of 91.2%. This indicates that the model maintained its flexibility to characterize new images and avoided overfitting to the limited training dataset. The shallow depth of the CNN, consisting of only three convolutional layers, further supports its generalizability^19^. Additionally, the fact that the validation loss was slightly below the training loss reinforces the absence of overfitting^20^.

The relatively high losses observed for both training and validation data indicate the challenge of accurately estimating the probabilistic output of the model compared to the factual binary labels assigned to each image^21^. While the model exhibited accurate predictions in most cases, there were instances where its output was incorrect, highlighting the need for further refinement. This observation suggests that enhancing the model's certainty, even in correct predictions, could be an area of focus for future improvements. An interesting observation was made when observing the relationship of training accuracy and validation accuracy over the course of the 64 epochs: After the 36th epoch, there was only one deviation to the general tendency of training accuracy being lower than testing accuracy, which was found at the 50th epoch where training accuracy achieved 86.92% and validation accuracy only achieved 86.81%. Similar to the pattern observed in accuracy, the validation loss became consistently lower than the training loss after approximately half of the training epochs. The last occurrence of the validation loss being lower was found in the 30th epoch; more specifically, the validation loss reached 0.3914, while the training loss reached 0.3407. This occurrence underscores the complexity of the validation set for the model during the earlier epochs. This temporary discrepancy may suggest specific characteristics of the validation set that proved challenging for the model, warranting further investigation.

When introducing the testing dataset, the model performed with a slightly lower accuracy of 93.41% than that of the validation dataset, along with a slightly higher loss value of 0.2209, which underscored the model’s accuracy. A recall value of 0.8148 suggested that the model often identified patients who did have lagophthalmos as positive, relative to frequency of false negative predictions. Furthermore, when the model did make a positive prediction, it was rarely a false positive, as demonstrated by the precision value of 0.9565. These two statistics translated into a high F1 score of 0.9697, reflecting a strong model accuracy. Meanwhile, the high specificity value of 0.9844 showed that the model was highly effective at determining when a patient did not have lagophthalmos. With an AUROC of 0.9627, the model also proved to have a strong ability to discriminate between positive and negative cases.

A similar study using artificial intelligence to diagnose ocular adnexa was capable of diagnosing periocular anatomy with an accuracy of 98.2%, reinforcing applicability of neural networks when diagnosing facial conditions^22^. This finding further supports the applicability of neural networks in facial condition diagnosis. However, it should be acknowledged that our study differs from previous works in terms of the specific diagnoses targeted and the use of still images rather than videos as input.

Moving forward, it would be valuable to extend this study by incorporating a larger training dataset. While we have demonstrated the generalizability of our model, further verification beyond this strong proof of concept is necessary before implementing the application in a clinical setting. The inclusion of more diverse images and additional patient cases can enhance the model's robustness and reliability, ensuring its effectiveness when deployed for real-world use. Future research may also compare the algorithm’s performance with novice human evaluators, as human evaluator panels represent a key testing tool in AI research^23^. Further, including additional races and ethnicities may improve the generalizability of our findings and help broaden the access to lagophthalmos care.

In conclusion, our study presents a novel application for comprehensive lagophthalmos diagnosis utilizing a CNN-based approach. This is the largest effort to automatize lagophthalmos diagnosis using AI-technology. The achieved results demonstrate the remarkable performance of the model in accurately diagnosing lagophthalmos-related periocular anatomy. By leveraging the power of mobile health solutions, our application offers the potential to improve clinical workflow, enhance diagnostic skills, prevent complications, enable continuous monitoring, and provide patient education and support. Further advancements and validation are crucial to ensure the safe and effective integration of this solution into routine patient care.

## Methods

From June 2019 to May 2021, prospective data acquisition was performed on 30 lagophthalmos patients seen at the Department of Plastic, Hand, and Reconstructive Surgery at the University Hospital Regensburg, Germany (IRB reference number: 20-2081-101). Informed consent was obtained from all subjects and/or their legal guardian(s) for study participation. Inclusion criteria involved patients who were at least 18 years of age, showed common symptoms of lagophthalmos, and were able to consent on their own behalf for care and study participation. Exclusion criteria included non-German-speaking and/or illiterate patients unable to give informed consent. Comparative data were gathered from 10 healthy patients as the control group. The photo documentation was performed by the first author (L.K.) using the CANON EOS 400D with the respective flash unit (Canon, Ota, Japan). Prior to patient enrollment, the first author underwent instruction training by the hospital’s photo documentation unit. All patient images were taken in one dedicated hospital room at the same spot to ensure standardized data collection. Distance between patient position and camera unit was 1.25 m. We used a camera tripod with fixed shot sizes to adjust the camera height (defined as central point of camera lens) to the patient’s facial center (defined as the midpoint between the nasion and the nasal tip). Examination room lighting was standardized at 1900 lx. As recommended by the Jena facial research group, patients were asked to perform the facial expressions to the best of their ability three times prior to photo documentation^24,25^. Overall, we obtained 1008 patient images which were used to train and evaluate a convolutional neural network (CNN). For an exemplary pathological image series, please refer to previous work by the Jena facial research group^26^. Please see Supplementary Material S1 for an exemplary physiological image series.

This study’s model and analysis was created in Python 3.7, using standard machine learning and data science libraries, including the Tensorflow, Matplotlib, and Numpy libraries. The final version of the CNN consisted of a customized architecture based on an AlexNet architecture. More specifically, this CNN was designed to be a so-called lightweight CNN, with three convolutional layers rather than five, in order to lower the network’s number of parameters. Furthermore, a dropout layer was introduced as a regularization technique to prevent overfitting, which is not present in AlexNet architecture. This specific architecture was optimized for an input image size of 256 by 256 pixels to avoid over-parametrization of the model. An 80:10:10 split between training, validation, and testing data was used, translating into 826 images for training, 91 pictures for validating the effectiveness of intermediate models, while 91 patient faces tested the final model’s accuracy. Moreover, recent research indicated that our dataset size was sufficient to create and test our classifier. In fact, a 2020 study used a dataset of 1000 red/green/blue (RGB) pictures, with a similar image resolution of 224 by 224 pixels, to develop robust three-layer CNN. Interestingly, the authors demonstrated that their image set was sufficient to include four image classes, while we limited our model to two image categories^27^. Based on these findings, our dataset size was deemed to be sufficient for the model’s development and testing. The CNN was trained over 64 epochs; a larger number of epochs was avoided in order to reduce the chances of overfitting. When the model’s tuning was completed, the testing set was used to evaluate the model that produced the highest validation accuracy in training. The model (Fig. 1) was created using rectified linear units (ReLUs) with each of the three convolutional layers. A “max-pool” layer was also placed between each convolutional layer. Finally, a ‘flatten’ layer transformed the output into a linear vector, and two ‘dense’ layers were used to associate the correlation between different image features with a certain output. It was ensured that the model’s weights were trained at no point on any form of testing data and that no synthetically generated training images were used, in order to preserve the complexity of dealing with realistic patient images and to avoid overfitting the model. The full source code is available at https://github.com/Siddysimon/lagonet.

### Ethical approval

All procedures performed in studies involving human participants were in accordance with the ethical standards of the institutional and/or national research committee and with the 1964 Helsinki Declaration and its later amendments or comparable ethical standards. The study was approved by the Bioethics Committee of the University of Regensburg (No. 20-2081-101).

## Conclusion

This study proposes a novel application for rapid and reliable lagophthalmos diagnosis. Our CNN-based approach combines effective anti-overfitting strategies, short training times, and high accuracy levels. Ultimately, this tool carries high translational potential to facilitate the physician’s workflow and improve overall lagophthalmos patient care.

### Supplementary Information

Supplementary Information.

## Data Availability

The datasets used and/or analysed during the current study are available from the author (Mr. Leonard Knoedler, Leonard.Knoedler@ukr.de) on reasonable request.
