# Supplementary material for: Diagnosing lagophthalmos using artificial intelligence
Source: Sci Rep. 2023 Dec 8;13:21657. doi: 10.1038/s41598-023-49006-3 (PMC10709577; doi:10.1038/s41598-023-49006-3)

**Supplementary Material 1.** Physiological facial image series based on the Jena protocol [25]. For an exemplary pathological image series, please refer to previous research work by the Jena facial research group.


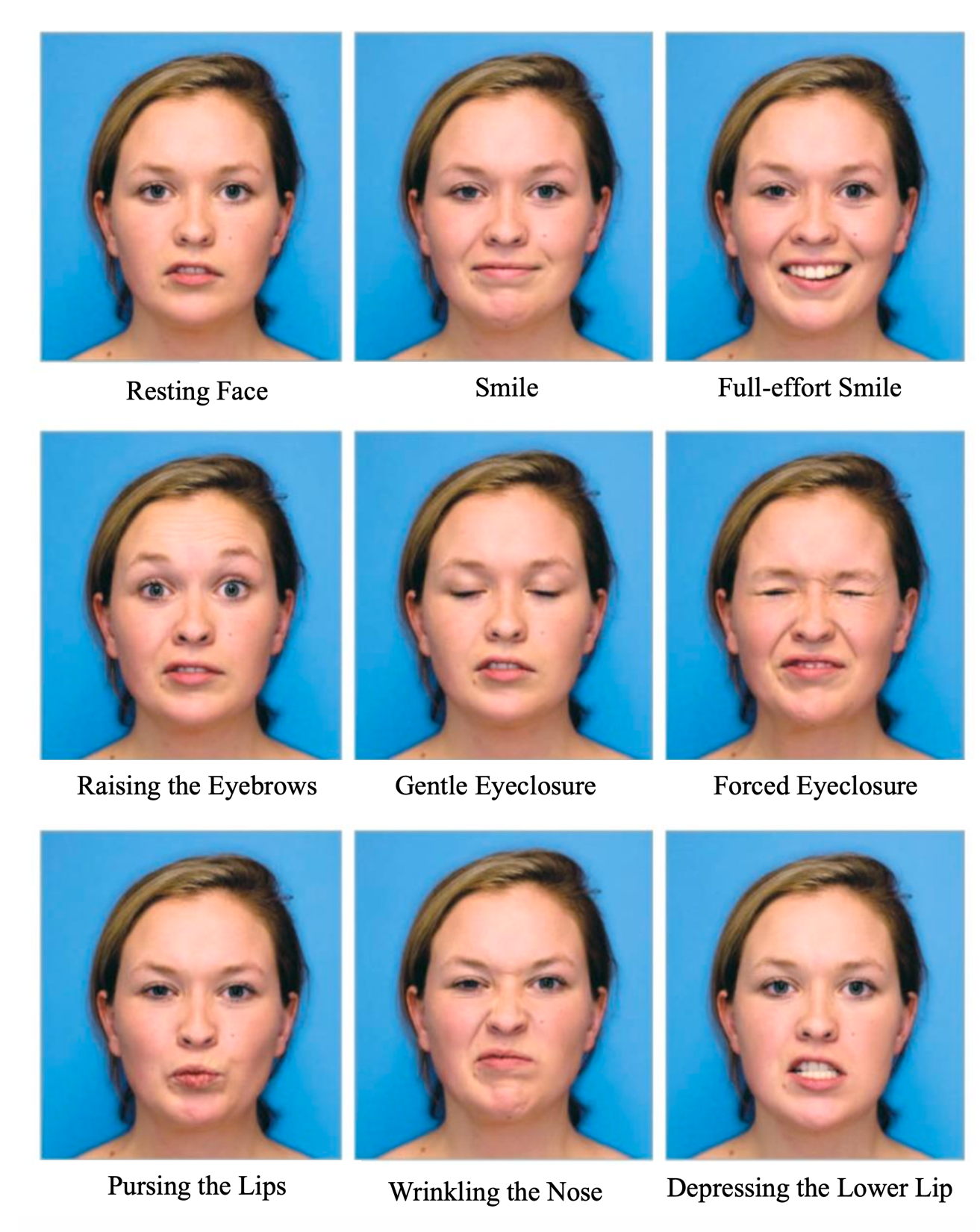

Supplement: Supplementary file 1 — Supplementary Information. [file 41598_2023_49006_MOESM1_ESM.docx]
